# Multiple courses of stereotactic re-irradiation in recurrent oligodendroglioma: a case report

**DOI:** 10.1186/1752-1947-5-183

**Published:** 2011-05-14

**Authors:** Shannon Fogh, Charles Glass, David W Andrews, Maria Werner-Wasik

**Affiliations:** 1 Department of Radiation Oncology, University of California San Francisco, 505 Parnassus Ave, Room L-08, Box 0226, San Francisco, CA 94143, USA; 2 Department of Radiation Oncology, Thomas Jefferson University, 111 South 11th Street, Philadelphia, PA 19107 USA; 3 Department of Neurosurgery, Thomas Jefferson University, 909 Walnut Street, 3rd Floor, Philadelphia, PA, USA

## Abstract

**Introduction:**

High grade gliomas are an insidious disease associated with an extremely poor prognosis. The role of re-irradiation for recurrent gliomas is unclear but several retrospective studies have indicated mild toxicity and modest outcomes with this regimen. With subsequent progression, it is unclear what options remain and more radiotherapy is rarely offered for fear of surpassing normal central nervous system tissue tolerance and causing significant side effects without significant benefit.

**Case presentation:**

In this report, we describe a 37-year-old Caucasian male initially diagnosed with a grade IV oligodendroglioma, who received multiple courses of re-irradiation and experienced a survival of 10 years with minimal cognitive or neurologic deficits.

**Conclusion:**

Significant toxicity with multiple courses of radiation does not always occur. Re-irradiation should be considered in a salvage setting.

## Introduction

The standard of treatment of newly diagnosed high-grade gliomas is resection followed by post-resection radiation therapy given with concurrent and adjuvant Temozolomide [1]. Recurrence is extremely common with limited treatment options [2]. There are many approaches currently available for the salvage treatment of patients with recurrent high-grade gliomas following initial radiation therapy including resection, re-irradiation or systemic agents but no standard of care exists. While practiced in some institutions, the role of re-irradiation for treatment of recurrence of disease is not well defined.

Reluctance to offer multiple courses of radiation stems from hesitation to exceed the radiation dose tolerances of normal tissue. Exceeding the dose that can typically be tolerated by a given structure can affect both short term and long term toxicity. As high grade gliomas generally recur within close proximity to the original location, maximum doses of radiation have typically been delivered to the area of recurrence. However, with improvement of imaging and radiation treatment techniques such as fractionated stereotactic radiation and the widespread availability of radiosurgery, we have the ability to deliver radiation with increased precision allowing irradiation to be delivered to the recurrent disease while decreased doses are delivered to the surrounding normal tissues [3-9].

Retrospective reviews and small randomized studies have indicated that re-irradiation to the tumor bed is feasible and may lead to improvement in survival with improved quality of life; however, offering multiple courses of radiation is rarely practiced [9,10].

In this report, we describe a case where four courses of irradiation were able to be delivered to different locations within the periphery of the tumor bed.

## Case presentation

Our patient was a 37-year-old Caucasian male who was initially diagnosed 12 years ago with a World Health Organization (WHO) grade IV oligodendroglioma of the right temporal lobe. He initially underwent resection and pathology was originally read as anaplastic oligodendroglioma. He was enrolled in RTOG 9402, a study which examined the effects of radiation alone versus pre-radiation chemotherapy for pure and mixed anaplastic oligodendrogliomas. However, on central review, pathology was reclassified as grade IV oligodendroglima and he was deemed ineligible for the study. He was subsequently treated with irradiation to a total dose of 60 Gy with concomitant procarbazine-lomustine-vincristine (PCV) chemotherapy. Disease progression was noted one year later in the tumor bed at which point a second resection was performed followed by a second course of fractionated stereotactic radiation therapy to a total dose of 35 Gy in 10 fractions.

**Figure 1:**
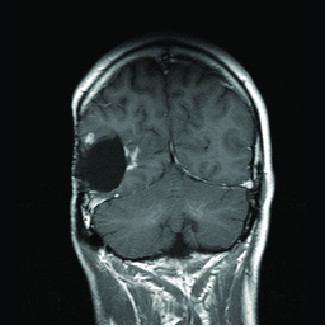
**MRI obtained for treatment planning prior to fourth course of radiation therapy**. Enhancement is noted adjacent to the surgical margin indicating progression of disease.

**Figure 2:**
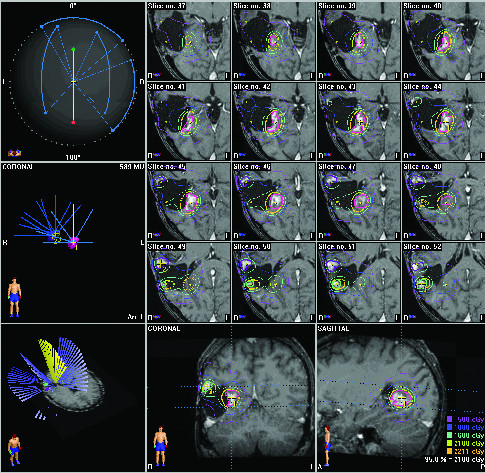
**Radiation treatment plan demonstrating targeting of peripheral tumor enhancement within the brain**.

Four years later, imaging indicated progression of disease in the tumor bed with nodular enhancement of the anterolateral margin of the surgical cavity in the right temporal region and he underwent radiosurgery to a total dose of 18 Gy given in one fraction. He developed a third recurrence the following year (see Figure 1) and the decision was made to treat three small enhancing lesions at the edge of his resection cavity (see Figure 2).

**Figure 3:**
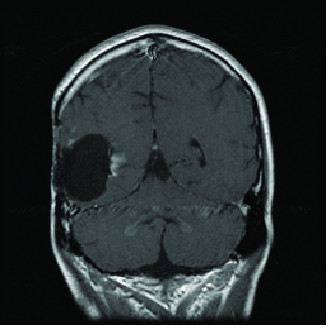
**MRI six months following fourth course of radiation therapy**. Improvement is shown in intensity of enhancement of the temporal lesion.

These three lesions, located in the medial posterior, lateral anterior and lateral posterior location around the surgical cavity, were treated with three separate isocenters and received doses of 21, 16 and 21 Gy respectively. The dose of 16 Gy was used for the lesion which had received 18 Gy the previous year. Follow-up Magnetic Resonance Imaging (MRI) completed six months following his fourth course of radiation therapy demonstrated improvement in intensity of enhancement of temporal lesion (see Figure 3).

Throughout his follow-up visits, his only complaints were intermittent headaches and seizures. Seizures were attributed to sub-therapeutic phenytoin, which resolved when switched to divalproex sodium. Later in his disease course he received 2 mg of daily decadron to control his headaches. He was able to achieve freedom from progression for over two years following his final course of radiation. His only neurological symptom occurred two months before his death and consisted of loss of peripheral vision in his left eye.

## Discussion

This case demonstrates that multiple courses of re-irradiation are feasible and may lead to improvement in quality of life and increased survival. Clinicians are reluctant to offer additional radiation therapy for recurrence both because of apprehension of exceeding normal structure tolerance as well as lack of evidence supporting this practice. Exceeding the dose that can typically be tolerated by a given structure can affect both short term and long term toxicity. This is particularly relevant when treating recurrent gliomas as tumors typically recur within close proximity to the original location where high doses of radiation have typically been delivered to the area of recurrence. In addition, the infiltrative nature of high-grade gliomas requires large margins when using standard external beam irradiation.

Both fractionated and single fraction stereotactic radiosurgery have been studied in re-irradiation of recurrent tumors. Stereotactic Radiosurgery (SRS) utilizes a steep dose gradient to deliver a highly conformal non-invasive single dose of radiation [4-6]. It is more commonly used for smaller treatment volumes and has also demonstrated reasonable median survival times after radiosurgery in very highly selected patients [5,7,8]. Radiation-induced necrosis in these studies was prevalent in studies where larger tumor volumes were treated.

Fractionated radiation therapy uses the same precision as radiosurgery but allows greater protection of normal structures while delivering an equivalent dose of radiation by delivering the dose over multiple treatment days. The largest study examining the efficacy and tolerability of fractionated radiation therapy consisted of 172 patients and demonstrated promising survival results with minimal rates of radiation induced side effects [9]. Other studies have also demonstrated similar survival rates with minimal toxicity in addition to improvement in neurological symptoms [10].

In our case, multiple courses of irradiation were able to be delivered following initial treatment in part because the residual areas to be treated were located at different positions along the periphery of the tumor that could be individually targeted (see Figure 3). While our patient was at risk for necrosis within the tumor bed, it is important to recognize that necrosis is considered a therapeutic effect of radiosurgery and the important component of treatment with respect to clinical outcomes is the sparing of normal tissue. By re-irradiating the recurrence at the edge of the tumor bed, we were able to treat the tumor recurrence and avoid normal tissue.

We acknowledge that the histopathologic grading of oligodendrogliomas is controversial and subject to interobserver variability. To the best of our knowledge, our patient was diagnosed with a WHO grade IV oligodendroglioma. Grade IV oligodendrogliomas essentially appear to be glial neoplasms with overwhelming features of glioblastoma multiforme (GBM) arising from known lower grade oligodendrogliomas or GBM with a significant proportion of oligodendroglial differentiation. The diagnostic utility of this diagnosis is uncertain as these tumors may behave either like glioblastoma or grade III oligodendrogliomas.

The updated WHO guidelines published in 2007 recommend classifying such tumors for the time being as 'glioblastoma with oligodendroglioma component'. It remains to be established whether or not these tumors carry a better prognosis than standard glioblastomas and we, therefore, chose to focus our case on the feasibility of delivering multiple courses of radiation rather than the prolonged survival of our patient.

## Conclusion

Multiple courses of re-irradiation are feasible and may lead to improvement in quality of life and increased survival in patients with high-grade gliomas. While the patient's age and histological diagnosis made his prognosis better compared to other patients with high-grade tumors, his extended survival was in part due to controlling his tumor with both surgery and multiple courses of irradiation.

This case illustrates the importance of individualizing care and maintaining a balance between the benefits and detriments of treatment. In the case of this patient, multiple courses could be delivered to a variety of areas along the periphery of the tumor bed as noted with minimal effect to the patient's well-being.

## Consent

Written informed consent was not obtained before the patient died and could not be obtained from the next of kin despite all reasonable attempts. All efforts have been made to protect the identity of the patient and there is no reason to believe that the family would object to publication. IRB approval was granted to review this case.

## Competing interests

The authors declare that they have no competing interests.

## Authors' contributions

SF participated in the design and drafted the manuscript. CG participated in the design and collection of the information. DA participated in the design and helped to draft the manuscript and MW-W participated in the design and edited the manuscript. All authors have read and approved the final manuscript.
